# Effect of COVID-19 Lockdown on Dietary Habits and Lifestyle of Food Science Students and Professionals from Spain

**DOI:** 10.3390/nu13051494

**Published:** 2021-04-28

**Authors:** Ricard Celorio-Sardà, Oriol Comas-Basté, M. Luz Latorre-Moratalla, María Fernanda Zerón-Rugerio, Mireia Urpi-Sarda, Montserrat Illán-Villanueva, Andreu Farran-Codina, Maria Izquierdo-Pulido, María del Carmen Vidal-Carou

**Affiliations:** 1 Departament de Nutrició, Ciències de l’Alimentació i Gastronomia, Facultat de Farmàcia i Ciències de l’Alimentació, Campus de l’Alimentació de Torribera, Universitat de Barcelona, Av. Prat de la Riba 171, 08921 Santa Coloma de Gramenet, Spain; ricard.celorio.97@gmail.com (R.C.-S.); oriolcomas@ub.edu (O.C.-B.); mariluzlatorre@ub.edu (M.L.L.-M.); fernanda.zeron@ub.edu (M.F.Z.-R.); murpi@ub.edu (M.U.-S.); millan@ub.edu (M.I.-V.); afarran@ub.edu (A.F.-C.); maria_izquierdo@ub.edu (M.I.-P.); 2 Federación Española de Dietistas-Nutricionistas Universitarios (FEDNU), C/Doctor Fleming, 53-PTA. 8. 46470 Albal, Spain; 3 Associació Catalana de Ciències de l’Alimentació (ACCA), Institut d’Estudis Catalans (IEC), C/del Carme 47, 08001 Barcelona, Spain; 4 Institut de Recerca en Nutrició i Seguretat Alimentària (INSA·UB), Universitat de Barcelona, Av. Prat de la Riba 171, 08921 Santa Coloma de Gramenet, Spain; 5 Xarxa d’Innovació Alimentària (XIA), C/Baldiri Reixac 4, 08028 Barcelona, Spain; 6 Associació Catalana de Científics i Tecnòlegs dels Aliments, Av. Prat de la Riba 171, 08921 Santa Coloma de Gramenet, Spain; 7 Centro de Investigación Biomédica en Red (CIBER) on Frailty and Healthy Ageing (CIBERFES), Instituto de Salud Carlos III, 28028 Madrid, Spain

**Keywords:** COVID-19, coronavirus, lockdown, dietary habits, lifestyle, e-survey, food sciences, college students

## Abstract

On 11 March 2020, the World Health Organization (WHO) declared COVID-19 a global pandemic, forcing countries around the world to confine their population to halt the rapid spread of the virus. This study aimed to evaluate the changes in dietary habits and lifestyle during the COVID-19 lockdown a specific population with academic and professional knowledge in food sciences from Spain. An online questionnaire, based on 41 items, including sociodemographic data, dietary habits, food-related behaviors, and lifestyle were distributed using academic and institutional mailing lists and social media. Results showed a higher intake of fruit and vegetables, legumes, eggs, fish, and yogurt together with a decrease in consumption of alcoholic beverages between before and during the lockdown period. Nevertheless, an increase in consumption of some fruitive foods and an increase in self-reported weight were also observed, although in lower percentages than in other populations. A worse sleep quality and an increase in working hours and sitting time were also reported. Overall, trends towards healthier dietary habits were observed within the study sample during COVID-19 confinement period.

## 1. Introduction

Coronavirus disease 2019 (COVID-19), caused by SARS coronavirus 2 (SARS-CoV-2), is an acute respiratory syndrome that emerged from Wuhan, China, in late 2019 [1]. On 11 March 2020, the World Health Organization (WHO) declared COVID-19 a global pandemic due to the widespread of newly reported cases and the rapidly increasing death toll [2], thus forcing countries around the world to confine their population to halt the spread of the virus. By the beginning of March 2021, more than 117M COVID-19 cases were reported worldwide and 3.15M specifically in Spain since the first diagnosed case in the country on 31 January 2020 [3].

During lockdown, countries developed various strategies to avoid mass gatherings by paying more attention to vulnerable populations, including people with underlying medical conditions or with an immune system compromised by a medical condition or treatment, health care providers, or older adults [4]. In Spain, the state of alarm was applied in mid-March to stop the uncontrolled transmission of the virus, an instruction that would force the total home confinement of the Spanish population, except for people working in essential activities (i.e., health professionals, security forces, emergency brigades, and food industry) [5]. The strict lockdown lasted until early June 2020 [6]. Most Spanish homes saw their income reduced due to temporary labor force adjustment plans while many others, including the whole academic community, had to promptly switch to teleworking. This led to many changes in lifestyle habits, especially those concerning food consumption and physical activity [7].

It is known that healthy eating and physical activity are key for health and well-being, especially when the immune system is challenged [8]. A healthy diet, based on plant-based food, healthy fats, and protein-rich food, together with weekly exercise and sunlight exposure, is set to help prevent viral diseases and enhance the human body in fighting infections such as COVID-19 [8]. However, sedentarism, unease, and tediousness caused by social isolation could lead to changes and worsening of lifestyle patterns while also promoting binge eating [9]. In this context, many scientific institutions, and societies from all over the world, such as the WHO or the “Sociedad Española de Nutrición Comunitaria” (SENC, Spanish Society of Community Nutrition), issued practical dietary guidelines and physical activity recommendations to help adapt the shopping list and the food intake of families to this new situation [10,11].

Recently, some studies have emerged worldwide intending to assess in detail how eating habits and lifestyle of people have changed during confinement, most of them showing general trends towards the adoption of unhealthy eating habits and weaker lifestyles [12,13,14,15,16,17,18,19,20,21,22]. Despite this, in Spain, a higher adherence to the Mediterranean Diet, which is well-known to be a healthy dietary pattern, was reported during lockdown in general population [23,24,25]. Nevertheless, in the study conducted by Sánchez-Sánchez et al., an increase in the consumption of alcoholic beverages, salty and fried snacks, and homemade confectionary was reported [23], as in the majority of studies on general population from other countries of the world, mentioned above.

However, there are still no studies that analyze whether these changes reported in the general population have also been adopted by a specific population subgroup with an expected knowledge in food, health, and lifestyle management, such as students and professionals of different disciplines of food science. To acknowledge the lockdown behavior of this collective would be important to assess in which extent their previous knowledge in nutrition and food sciences could influence their choices. Hence, the current study aimed to detect any change in dietary habits and lifestyle of a specific sample of Spanish students and professionals of food science during the COVID-19 lockdown period. Thus, specific information on dietary habits, food-related behaviors, and other lifestyle variables of this population were evaluated to confirm the hypothesis that a background in nutrition and food sciences encourages citizens to make healthier choices, even in adverse situations, such as this period of confinement.

## 2. Materials and Methods

### 2.1. Study Design and Participants

This is a cross-sectional study with convenience sampling based on an online survey conducted using Google Forms web survey platform. The link to the online survey was shared through academic mailing lists of the Food and Nutrition Torribera Campus of the University of Barcelona, and through social media and mailing lists of students and professional organizations. Specifically, the survey was shared within members of the “Federación Española de Dietistas-Nutricionistas Universitarios” (FEDNU, Spanish Federation of Nutrition and Dietetics Students), the “Associació Catalana de Científics i Tecnòlegs dels Aliments” (Catalan Association of Food Scientists and Technologists) and the “Associació Catalana de Ciències de l’Alimentació” (ACCA, Catalan Association of Food Sciences). Participants were also asked to share the link to the survey with their professional colleagues. Participation in the online survey was entirely voluntary and anonymous. Participants were informed about the purpose of the research and asked for permission to use and publish the data from the study before starting the questionnaire.

The questionnaire was available online from 22 May to 3 July 2020 in two language versions, Catalan and Spanish, with the same design and set of variables. A total of 339 participants completed the survey. After data depuration (i.e., missing data and duplicate answers), the final data set included 321 participants.

### 2.2. Questionnaire

Data were collected through an online questionnaire containing 41 items divided into 4 sections designed to assess: sociodemographic data, frequency of food consumption and culinary habits, general eating habits, and lifestyle of the participants. The study was conducted in agreement with the Declaration of Helsinki, and all data were collected and recorded according to the Spanish Organic Law on Protection of Personal Data and Guarantee of Digital Rights (LOPD-GDD) 3/2018. A translated version of the questionnaire can be found in Table S1.

#### 2.2.1. Sociodemographic Information

In this section, 10 items regarding sociodemographic data were included: gender, age, self-reported height, and weight (usual and during lockdown), place of residence, home cohabitants, marital status, collective to which they belonged to (student or professional) and the type of studies they had followed or were following at the time (i.e., human nutrition and dietetics, food science and technology, culinary sciences, or others).

#### 2.2.2. Frequency of Food Consumption and Culinary Habits

In the second section of the questionnaire, participants completed a multiple-choice grid designed to assess food consumption before and during confinement. It was divided into 5 parts (i.e., fruit and vegetables, protein-rich food, eggs and dairy products, carbohydrates, snacks, and drinks) with a total of 30 items. Respondents could choose the frequency of consumption of each item twice (before and during lockdown) among never consumed, sporadically (once or less a month), twice to three times a month, once a week, twice to three times a week, four to six times a week, every day, and several times a day. From these data, the percentage of the population that reported a change (increase or decrease) in food intake was calculated.

Furthermore, questions regarding culinary habits such as fat used for cooking, number of meals of the day and time of meals, before and during confinement, were also included in this section.

#### 2.2.3. General Eating Habits

To evaluate the eating behaviors of the population studied during lockdown, participants were asked whether they had changed their eating patterns or had begun to follow a vegan or vegetarian diet. In addition, the perception of weight changes since the beginning of confinement was included.

Likewise, culinary practices of participants, as well as consumption of fried foods, water, coffee and tea, alcoholic beverages, and the use of food delivery during confinement was assessed using a Likert scale, with 0 being totally disagree and 5 completely agree. Additionally, a question was included at the end of the section asking whether participants were taking any kind of nutritional supplement.

#### 2.2.4. Lifestyle

In the last section of the questionnaire, a set of questions was asked to evaluate the lifestyle behaviors of participants during confinement, including physical activity, sedentary activities, sleep parameters, sunlight exposure, and tobacco smoking.

The following questions were used to evaluate the lifestyle habits of the participants: “How often do you perform at least 30 min of physical activity before and during confinement?”, with answers ranging from “once a week” to “daily“; “How many hours do you spend on a daily basis doing sedentary activities before and during confinement?” and answers ranging from “<1 h” to “≥8 h”; “How would you rate the quality of your sleep during confinement?” with answers ranging from “better than before confinement” to “worse than before confinement”; “During confinement, my bed/wake-up time was…” with answers comprising from “earlier than before confinement” to “later than before confinement”; “How often were you exposed to sunlight during confinement?” with answers ranging from “never” to “daily” and “Do you smoke?” and in case of affirmative answer, “If so, during confinement, do you smoke less, the same, or more than before?”.

### 2.3. Statistical Analysis

Distribution and normality of data was assessed through Shapiro–Wilk. Differences in food frequency consumption before and during lockdown were tested using nonparametric paired-sample Wilcoxon test for the whole sample and for each collective. To test whether changes in dietary habits were associated with physical activity patterns Chi-Squared tests were performed. General lineal models were conducted to test the association between food consumption and age and collective variables. Subsequently, Tukey post-hoc tests were used to compare the differences. Values of *p* < 0.05 were accepted as significant. All statistical analyses were performed using SPSS for Windows, version 22 (Chicago, IL, USA).

### 2.4. Visualization Tools

A heatmap was constructed using *Metaboanalyst 5.0* as a visualization tool [26]. It was created using the average differences in food consumption frequency before and during lockdown and stratifying the population in 5 collectives: students of BSc in Human Nutrition and Dietetics (HND), of BSc in Food Science and Technology (FST), of BSc in Culinary and Gastronomic Science (CGS), of MSc or PhD in food-related areas, and Food Sciences Professionals. This heatmap has been used to better visualize the magnitude and direction of changes in food intake.

## 3. Results and Discussion

### 3.1. Socio-Demographic Data of the Study Population

Briefly, of the total 321 total respondents, the majority were females (79.8%) and aged between 18 and 25 years old (67%) (Table 1). This gender distribution reflects the actual nature of a professional field with a female predominance. A high percentage of polled individuals declared living in their habitual residence during the lockdown period, within a familiar environment. In fact, only a 6.2% were living alone or with friends.

The collective of students of nutrition and food sciences was the largest sample subgroup, with 73, 8% of the total sample, followed by professionals and professors. Regarding the academic background, undergraduates were the most prevalent respondents. Specifically, 51% were enrolled in a BSc in HND, 27% in a BSc in FST and 14% in a BSc in CGS. The rest were postgraduate program students, such as MSc or PhD in food-related areas.

### 3.2. Effect of Lockdown on Dietary Habits

**Figure 1:**
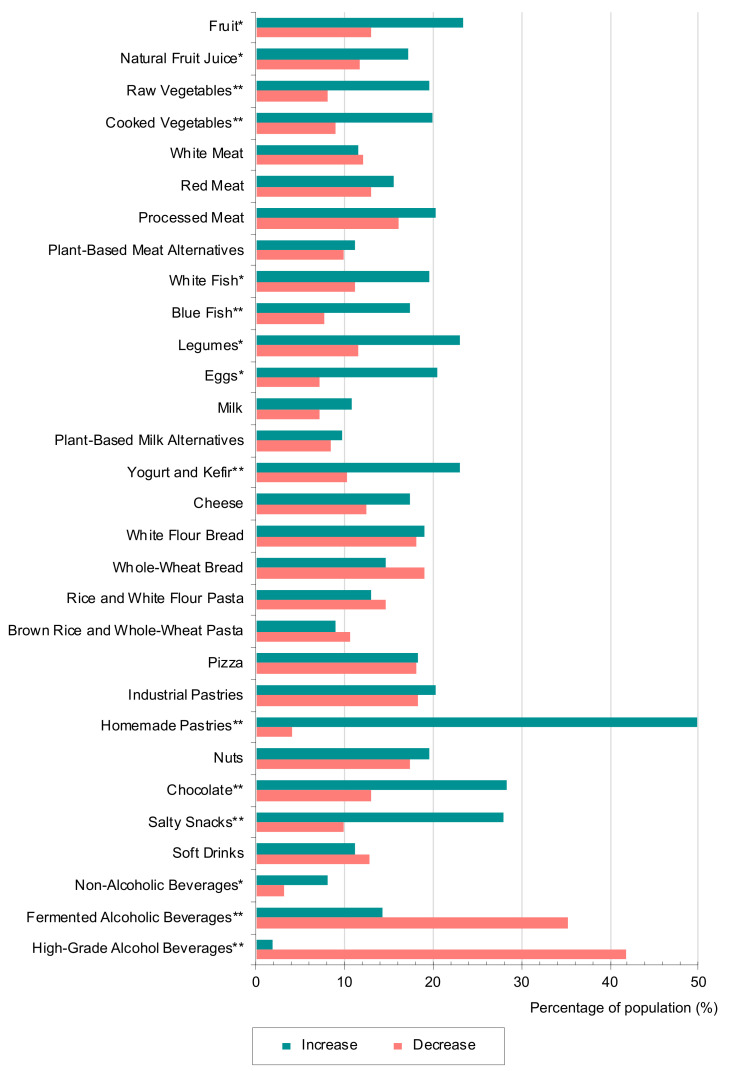
Percentage of population that reported a variation (increase or decrease) in the intake of each foodstuff during COVID-19 lockdown. People who reported no change in frequency of consumption are not represented in this figure. Statistically significant differences between food intake before and during COVID-19 lockdown period were assessed by a Wilcoxon test and are indicated with asterisks (* *p* < 0.05; ** *p* < 0.01).

**Figure 2:**
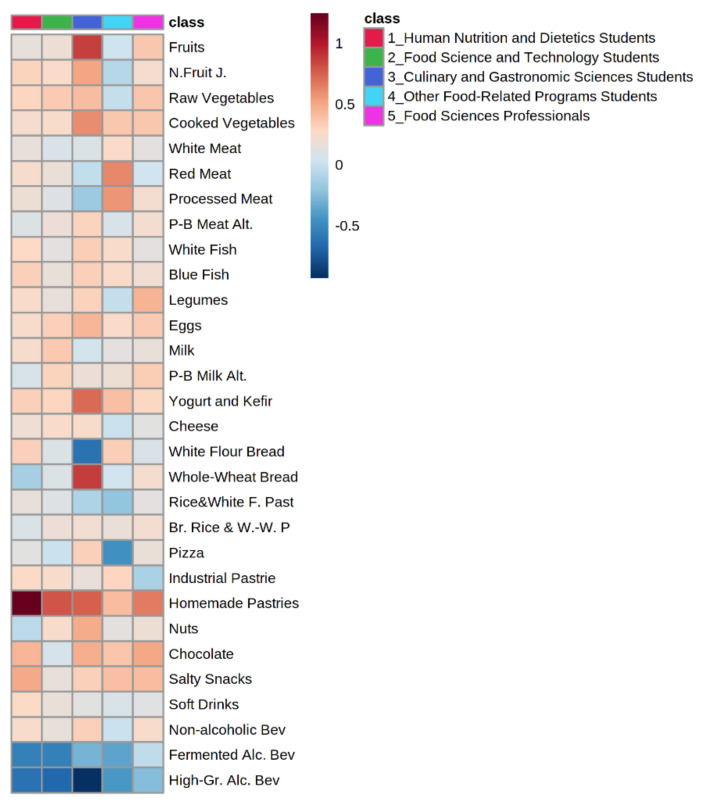
Heatmap visualization showed the magnitude of the variation (increase or decrease) in the intake of each foodstuff during COVID-19 lockdown considering the different collectives^1^ of the study sample. Blue color scale indicates a decrease in consumption and red color scale corresponds to an increase in consumption. ^1^Number and percentage of individuals by collective (*n*, %): Human Nutrition and Dietetics Students (122, 38%), Food Science and Technology Students (64, 20%), Culinary and Gastronomic Sciences Students (33, 10%), Other Food-Related Programs Students (18, 6%), Food Sciences Professionals (84, 26%).

Frequencies of consumption of all the reviewed foodstuff, both before and during lockdown, is displayed as Supplementary Material (Table S2). As shown in Figure 1, a significant increase in the frequency of consumption of fruit, vegetables, and legumes (*p* < 0.01) was observed. Regarding animal-origin foods, an increased intake frequency was only reported in fish (both non-fatty and fatty fish), eggs and yogurt (*p* < 0.01). The percentage of population that reported an increase in consumption ranged between 17% and 23% in all these items, all of them associated with a healthy dietary pattern [27]. When stratifying by academic background in the collective of students, this increase was especially important in students of the BSc in HND, as seen in Figure 2.

Data obtained from food science students and professionals are consistent with data from similar studies conducted in general population from Spain [24,25]. According to these other studies, the consumption of fruit, vegetables, eggs, and legumes was reported to increase by 25% of the population during the lockdown period. However, in almost all studies performed in other European countries, and in the rest of the world, the consumption of these healthy foodstuffs did not show this growing trend, unlike the consumption of fried food, sweet and salty snacks, pizza, and pastries [12,13,14,15,16,17,18,19,20,21,22]. The higher fruit and vegetable intake found in this study could be partially explained by the fact that production of fruits in Spain was higher than other EU countries [28], and they were more accessible to everyone as their prices are lower than in other countries [29].

A rise in consumption of some fruitive foods, which are consumed for pleasure rather than for its nutritious purpose, was also observed in this study. Concretely, the greatest increase was reported for homemade pastries (50%), followed by chocolate and salty snacks (28% each). It is worth mentioning that the increase in the consumption of these foods, recommended to be just occasionally consumed, was higher than the obtained for the healthier foodstuffs mentioned above. At a Spanish level, Sánchez-Sánchez et al. [23] and Rodríguez-Pérez et al. [25] also found an increase in the consumption of these products. Specifically, it is noteworthy that the significant increase observed in homemade pastries consumption, occurred in both Spanish populations and throughout the world [15,17,23,25]. Conversely, Pérez-Rodrigo et al. [24] reported a decrease in consumption of pizza, chocolate, and sweetened beverages, especially in the population over 55 years old.

Non-alcoholic beverages category showed a slight, but statistically significant, increase during the lockdown period (*p*-value = 0.025), with 8% of the population reporting this trend. This is mainly due to higher consumption of water, provided that coffee and tea reported trends towards a decrease in their consumption. In fact, 56.7% of the study sample reported a decrease in coffee and tea consumption during confinement.

The only statistically significant decrease was reported in consumption of both fermented and high-grade alcoholic beverages, with 35% and 42% of the population reporting a reduction, respectively. In contrast, several studies conducted in general population worldwide have reported a significant increase in the consumption of alcoholic beverages [12,13,14,15,16,17,18,19,20,22,23,24,25]. This could be explained by the fact that the sample of the current study is mainly composed by students, a population subgroup that generally links alcohol consumption with leisure activities, which were highly restricted in this period of time [24]. Indeed, when stratifying for collectives, professionals from the food science field showed a minor change for fermented alcoholic beverages, while HND and FST BSc students reported a significantly higher decrease. Regarding high-grade alcoholic beverages, a significant decrease was reported in all the collectives (*p* < 0.05) (Figure 2). It is necessary to highlight that the collective of BSc CGS students changed the use of white bread for whole-wheat bread during lockdown and the increase in fruit consumption. Moreover, it was observed that, in general, all collectives had significantly increased the consumption of homemade pastries during this period (*p* < 0.05).

Figure Abbreviations: N.Fruit J., Natural fruit juices; P-B Meat Alt., Plant-Based Meat Alternatives; P-B. Milk Alt., Plant-Based Milk Alternatives; Rice-White F. Pasta, Rice and White Flour Pasta; Br. Rice – W.-W. Pa, Brown Rice and Whole-Wheat Pasta; Indust. Pastries, Industrial pastries; Non-alcoholic Bev, Non-alcoholic Beverages; Fermented Alc. Bev., Fermented Alcoholic Beverages; High-Gr. Alc. Bev., High-Grade Alcoholic Beverages.

By comparing the behavior during lockdown amongst collectives, only the decrease in the frequency of consumption of high-grade alcohol beverages (*p* = 0.03) and the increase in fruit (*p* = 0.01) and homemade pastries (*p* = 0.02) were significantly associated with the belonging collective, following the trends observed in the heatmap. Furthermore, results from the post-hoc analysis showed that HND students responded a higher increase in homemade pastries consumption than professionals of the food science field, while CGS students reported a larger significant decrease in alcohol than the other collectives, especially professionals, during the lockdown period. On the other hand, when the association between age and food consumption was analyzed, it was observed that only changes in the consumption of alcoholic beverages (i.e., fermented and high-grade) were statistically significant (*p* < 0.01). Specifically, respondents aged 18 to 25 years (mainly students) significantly had a greater reduction in alcohol consumption compared to the others. However, a subsequent general lineal model including both variables (age and collective of respondents), resulted in no significant interactions with any of the foodstuff consumption reviewed in this study.

### 3.3. Effect of Lockdown on Food-Related Behaviours

Regarding meal frequency, it was observed that 7.2% of the participants skipped breakfast during lockdown, while lunch and dinner were unanimously maintained during the lockdown period. Moreover, 45.6% of the sample reported to stop having a midmorning snack while 23.4% reported to start having an afternoon snack and 53.7% a late-night snack before going to sleep. The introduction of a late-night snack has also been reported in other studies [13,16], as well as the maintenance of the three main meals of the day [12,15,24].

On meal timing, respondents reported to have had their first and last meal of the day at a time significantly later than before confinement. The mean time of the first meal before lockdown was 8:20 am (±1:54 h) and during lockdown was 9:34 am (±2:01 h). For the last meal, the mean time before and during lockdown was 9:30 pm (±1:14 h) and 9:47 pm (±1:35 h), respectively. Both meals experienced a statistically significant delay during confinement (*p* < 0.05).

**Figure 3:**
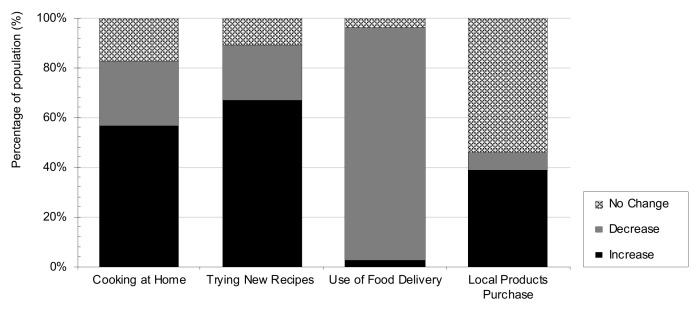
Percentage of population that reported changes in culinary behaviors during COVID-19 lockdown.

Concerning culinary behaviors during the lockdown period (Figure 3), 57% of the samples reported having increased their home cooking practices, and 67% tried elaborate new recipes. Pérez-Rodrigo et al. [24] also reported an increase in home cooking during confinement, although at lower rates (14%). The difference between those two percentages could be partially explained by the fact that our population, mainly students, was not used to cooking at home on a daily basis. No different trends between students and professionals were observed in these behaviors. Additionally, during lockdown, 39% of people reported a greater purchase of local products than before, and 94% of the sample reported a decrease in the use of food delivery from restaurants to their homes, in contrast with the 30% increase in food delivery services in The Netherlands [30].

When asked if the kind of fat used for cooking was changed during the lockdown period, 86.6% of the total sample responded negatively. Of those who answered affirmatively (5.9%), 86.1% reported an increase in extra virgin olive oil use for cooking, and also, 23.3% reported a decrease in butter and margarine use during the lockdown period, showing trends towards the adoption of a healthier lifestyle, together with the increase in fruit, vegetables, and fish consumption mentioned above.

Among the study population, 16.8% declared to follow a vegetarian or vegan diet. It is also noteworthy that another 7.2% adopted this kind of diet during the lockdown period and that 3.4% stopped following it. These data indicate that the percentage of people following a plant-based dietary patterns within students and professionals in the food science area is considerably higher than the 7.8% reported by Lantern Consultant for the Spanish population over 18 years old [31].

16% of the sample reported the intake of some type of nutritional supplement during the lockdown period, mainly protein and multi-vitaminic supplements. It is worth highlighting that those respondents studying the BSc in HND were the sample group who answered the most affirmatively to this question (28%). Another Spanish study also found that 14.5% of general population reported taking dietary supplementation during this period [24].

Data on food safety-related practices showed that 48% of the sample treated some of their food differently at home by cleaning and disinfecting it with bleach or other sanitizing products from the start of the lockdown period. Another 18% of the sample also reported such practices but claimed to have applied them sporadically. Moreover, 14.6% of the sample had to always quarantine the food after its acquisition. It is worth mentioning that it has been reported that the use of these products on food and the risk of ingestion and consequent toxicity from improperly stored hand sanitizers, cleaners, and disinfectants is considerable among the general population who normally has a gap in knowledge about safe preparation, use, and storage of cleaners and disinfectants [32].

Regarding weight management perception, 32% of the sample reported an increase in weight during confinement, while 19% reported some kind of decrease in their weight. Although in lower percentages, these findings are consistent with other studies that also indicated higher self-reported weight in populations from Spain (37.3%) [23], Italy (48.6%) [16], Chile (38.1%) [12], and France (35%) [33]. It would be interesting to study if this difference would be significant between the food science collective and general population and which percentage of general population had the perception of decreasing their weight.

It is worth highlighting that the percentage of people who reported an increase in their weight during confinement in this research is lower than that in studies performed on other populations. This could be explained by the knowledge that our sample has in terms of eating behavior management. Because, although showing similar trends in frequency of food consumption, a higher percentage of people still managed to maintain, or even decrease, their body weight during confinement.

### 3.4. Effect of Lockdown on Lifestyle

The impact of COVID-19 lockdown period on lifestyle was assessed by specifically reviewing sedentarism and physical activity, sleep parameters (timing and quality), as well as exposure to sunlight and tobacco smoking habits of the study population during confinement.

**Figure 4:**
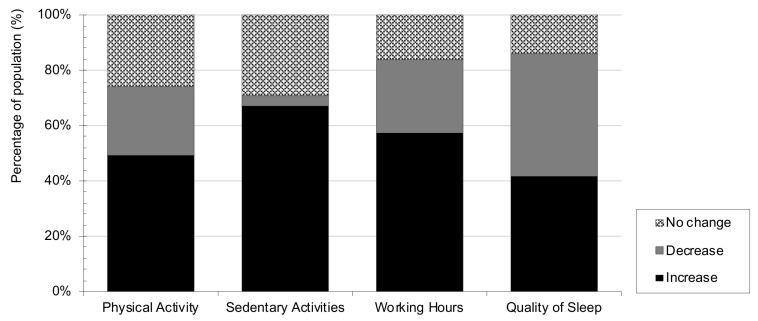
Percentage of population that reported changes in lifestyle habits during COVID-19 lockdown.

During the COVID-19 lockdown period, the population was asked to stay at home and work from home as well. Therefore, an increase in sedentarism was expected, which is consistent with the findings of the current study. In fact, 67.3% reported an increase in sitting time and only 3.7% reduced it during lockdown (Figure 4). Moreover, it was found that 31.2% of the sample reported spending 8 h or more doing sedentary activities such as studying, working, reading, watching television, or checking social networks. These findings in students and professionals from the food science field are consistent with those observed in other general population studies, where the majority of respondents reported being less active during the confinement period [24,25,34,35]. Regarding working hours, 57% of the sample reported having spent more time working during confinement than before (Figure 4). When asked about physical activity, 49.4% of respondents showed an increase in the frequency with which they practiced at least 30 min of exercise during confinement (Figure 4). Specifically, 20.5% of the sample reported practicing at least 30 min of exercise every day while, on the other hand, 13.7% reported to practice sport less than one day per week during the lockdown period. According to Sánchez–Sánchez et al., 15% of the adult Spanish population reported not practicing sport at all during COVID-19 lockdown [24].

It was also observed that those who increased their physical activity during lockdown also increased the intake of healthy foods, such as fruit (*p* < 0.01), raw and cooked vegetables (*p* < 0.01), and plant-based milk alternatives (*p* < 0.01) while decreased the frequency of consumption of white bread (*p* = 0.02), white rice, and pasta (*p* < 0.01), and industrial pastries (*p* = 0.02). These findings suggest that improvements in dietary habits are often accompanied by healthier lifestyles, as in our study, and in physical activity.

Consistent with other studies, it was observed that 44.5% of the participants reported having poor sleep quality during lockdown (Figure 4), which could be associated with greater anxiety and stress [24,36,37,38]. Furthermore, data showed that more than half of the population studied delayed their bed (54.1%) and wake-up timing (65.9%) during lockdown, which is also in agreement with other studies [39,40]. In this regard, Blume et al. [38] postulated that this delay in bed and wake-up timing could be associated with greater flexibility in working hours.

Within the food science collective considered in the current study, only 10.9% reported spending at least 15 min per day under the sunlight during the lockdown, in accordance with general health recommendation. In fact, 24.9% reported to have never followed this practice. On this topic, it has been reported that exposure to sunlight was one of the factors that people missed and affected their psychological status the most during lockdown [19].

Considering smoking habits, 12.5% of the study population declared to be smokers, a notably lower percentage than the general population (22%) according to the most recent Spanish National Health Survey [41]. Of them, 22% reported to have smoked more during lockdown, in contrast to the 30% that decreased this practice. It is noteworthy that 6 subjects reported to have quit smoking during the lockdown period, more than that found by Di Renzo et al. in Italian population [15].

## 4. Conclusions

This study focused on evaluating changes in dietary habits and lifestyle during the COVID-19 lockdown in Spain, targeting a specific population with academic and professional knowledge in food sciences. It is concluded that this specific population has overall adopted healthier dietary habits during confinement, that is, a higher intake of fruit and vegetables, legumes, eggs, fish, and yogurt together with a decrease in consumption of alcoholic beverages, compared with their previous behavior. However, despite the specific knowledge in food sciences that can be attributed to the study population, similar trends were observed in comparison with Spanish general population yet consuming slightly higher percentages of certain healthy foods. Nevertheless, this collective also reported higher consumption of fruitive foods (i.e., salty snacks, chocolate, and homemade confectionary) as it was observed for general population of the rest of the world.

Regarding food-related behaviors, a significant increase was observed in home cooking and local products acquisition for the targeted population. Additionally, a third of the population stated an increase in their self-reported weight, although it is a lower percentage than in other populations. Finally, the higher rate of sedentarism and poor sleep quality, reported in previous studies, were also reflected in this food science-centered population.

This study has certain limitations, starting with its cross-sectional nature, that does not allow us to find causality as well as the use of self-reported questionnaires that are prone to underreporting. Another limitation is the fact that changes in diet and lifestyle habits were assessed before and during lockdown using a single questionnaire, which can lead to a certain recall bias. Furthermore, it is recognized that the study sample is not representative of the entire population and therefore, the conclusions cannot be extrapolated. However, the main goal of this study was to assess dietary and lifestyle changes in subjects with knowledge in the field of nutrition and food science.

Overall, it would be desirable that the improvement in eating habits observed during COVID-19 lockdown could be maintained throughout the time as it could have a positive impact on the prevention of chronic diseases and COVID-19-related complications. A correction in those trends that are less desirable, such as increased consumption of fruitive foods and sedentary patterns, would also be advisable.

## Figures and Tables

**Table 1:** Socio-demographic variables of the study population.

Variables	All; *n* (%)	Female; *n* (%)	Male; *n* (%)
	321 (100)	256 (79.8)	65 (20.2)
**Age (intervals)**			
18–25	215 (67.0)	185 (72.3)	30 (46.2)
26–35	35 (10.9)	30 (11.7)	5 (7.7)
36–55	39 (12.1)	25 (9.7)	14 (21.5)
56–65	26 (8.1)	15 (5.8)	11 (16.9)
>65	6 (1.9)	1 (0.5)	5 (7.7)
**Collective**			
Student	237 (73.8)	198 (77.3)	39 (60.0)
Professional	84 (26.2)	58 (22.7)	26 (40.0)
**Marital Status**			
Single	211 (65.7)	181 (70.7)	30 (46.2)
Married	41 (12.8)	23 (9.0)	18 (27.7)
Living as Couple	51 (15.9)	38 (14.8)	13 (20.0)
Divorced	8 (2.5)	4 (1.6)	4 (6.1)
Widowed	1 (0.3)	1 (0.4)	0 (-)
Other	9 (2.8)	9 (3.5)	0 (-)
**Living in Habitual Residence ^1^**			
Yes	277 (86.3)	217 (84.8)	60 (92.3)
No	44 (13.7)	39 (15.2)	5 (7.7)
**People in Residence ^1^**			
Alone	9 (2.8)	5 (2.0)	4 (6.2)
Couple and Children	102 (31.8)	78 (30.5)	24 (36.9)
Parents and Siblings	142 (44.2)	124 (48.4)	18 (27.7)
Couple	49 (15.3)	35 (13.6)	14 (21.5)
Friends	11 (3.4)	9 (3.5)	2 (3.1)
Other	8 (2.5)	5 (2.0)	3 (4.6)

^1^ During the lockdown period (March–June 2020).

## Supplementary Materials

The following are available online at https://www.mdpi.com/article/10.3390/nu13051494/s1, Table S1: Questionnaire, Table S2: Percentage of the sample that reported the frequency of consumption, before and during confinement, for each foodstuff.
