# Supplementary material for: Effect of COVID-19 Lockdown on Dietary Habits and Lifestyle of Food Science Students and Professionals from Spain
Source: Nutrients. 2021 Apr 28;13(5):1494. doi: 10.3390/nu13051494 (PMC8146598; doi:10.3390/nu13051494)
Supplement: Supplementary file 1 [file nutrients-13-01494-s001.zip › nutrients-1181144-supplementary.pdf]

Table S1. Questionnaire

Questions	Answers
**SOCIODEMOGRAPHIC DATA**	
Gender	Woman / Man / Other
Age range	18-25 / 26-35 / 36-55 / 56-65 / >65
Height	Open Answer
Current weight	Open Answer
Collective	Student / Professional
If student, which study program	BSc in HND/ BSc in FST / BSc in CSG / Postgraduate
Province of residence	Open Answer
Marital status	Single / Married / Living as a Couple / Divorced / Widowed / Other
Living in habitual residence	Yes / No
Cohabitants	Alone / Couple & Children / Parents & Siblings / Couple / Friends / Other
**FREQUENCY OF FOOD CONSUMPTION AND CULINARY HABITS**	
Fruit<br>Natural fruit juice<br>Raw vegetables<br>Cooked vegetables<br>White meat<br>Red meat<br>Processed meat<br>Plant-based meat alternatives<br>White fish<br>Fatty fish<br>Legumes<br>Eggs<br>Milk<br>Plant-based milk alternatives<br>Yogurt or kefir<br>Cheese<br>White bread<br>Whole-wheat bread<br>Rice, pasta and potatoes<br>Whole-wheat pasta and cereals<br>Pizza<br>Industrial pastries<br>Homemade pastries<br>Nuts<br>Chocolate<br>Salty snacks<br>Sweetened and Soft drinks<br>Non-alcoholic beverages (water, tea, coffee)<br>Fermented alcoholic beverages<br>High-grade alcoholic beverages	Never / ≤ 1 a month / 2-3 a month / 1s week / 2-3 a week / 4-6 a week / Every day / >1 a day
Have you changed the fat used for cooking during confinement?	Yes / No / Sometimes

If affirmative, in which way	More extra virgin olive oil / Less extra virgin olive oil / More olive oil / Less olive oil / More seed oils / Less seed oils / More butter or margarine / Less butter or margarine
Which meals do you usually take?<br>Breakfast<br>Mid-day snack<br>Lunch<br>Mid-afternoon snack<br>Dinner<br>Late-night snack	Before Confinement / During Confinement
At what time did you usually take the first meal of the day before confinement?<br>At what time did you usually take the last meal of the day before confinement?<br>At what time did you usually take the first meal of the day during confinement?<br>At what time did you usually take the last meal of the day during confinement?	Open Answer
**GENERAL EATING HABITS**	
Do you think you have gained weight during confinement?	Yes / No / No change / DK
Did you follow a vegetarian/vegan diet during confinement?	Yes, always / Yes, I started now / No, I quitted now / No, never
During confinement I have cooked more than before<br>During confinement I have often tried to make new recipes<br>During confinement I have eaten more fried foods<br>During confinement I have used food delivery services more than before<br>During confinement I have drunk more water than before<br>During confinement I have drunk more tea or coffee than before<br>During confinement I have drunk more alcoholic beverages	Totally disagree 1 – 5 Totally agree
Have you taken some kind of dietetic supplement during confinement?	Yes / No
If affirmative, which one?	Open Answer
**LIFESTYLE**	
How many times a week have you exposed yourself to sunlight for at least 15 minutes?	Never / 1-3 days / 4-6 days / Every day
How often do you do at least 30 minutes of exercise or sport…<br>…Before confinement?<br>…During confinement?	< 1s a week / 1s a week / 2-3 days a week / 4-6 days a week / Every day
In total, how much time a day do you spend on sedentary activities…<br>…Before confinement?	< 1h / 1-2h / 3-4h / 5-7h / ≥8h

…During confinement?	
Do you usually smoke?	Yes / No
If affirmative, during confinement you smoke…	More than before / The same / Less than before / I quitted / I started
In relation with your sleeping habits, during confinement…	
On weekdays I wake up	Earlier than before / Later than before / At the same time
On weekdays I go to sleep	
On weekends I wake up	
On weekends I go to sleep	
During confinement, my sleep quality is…	Better than before / The same / Worse than before
During confinement, the number of hours dedicated to your work or student activity is...	Higher than before / The same / Lower than before
During confinement I try to purchase more local products	Yes / No / The same
During confinement I have changed the way I treat food after buying it	Yes / No / Sometimes
During confinement, I leave some food in quarantine before consuming it	Yes / No / Sometimes

Table S2. Percentage of the sample that reported the frequency of consumption, before and during confinement, for each foodstuff.

<table>
<tr><th>Foodstuff</th><th>Never</th><th>≤ 1/month</th><th>2-3/month</th><th>1/week</th><th>2-3/week</th><th>4-6/week</th><th>Every day</th><th>>1/day</th></tr>
<tr><td>Fruit</td><td>0.9 | 0.9 *</td><td>4.4 | 3.4</td><td>2.5 | 1.9</td><td>5.9 | 3.4</td><td>8.7 | 10.3</td><td>13.1 | 10.9</td><td>23.4 | 27.4</td><td>41.1 | 41.7</td></tr>
<tr><td>Natural Fruit Juice</td><td>33.0 | 36.1</td><td>28.7 | 24.6</td><td>13.1 | 9.3</td><td>5.9 | 7.2</td><td>8.4 | 9.0</td><td>4.4 | 4.7</td><td>5.6 | 7.8</td><td>0.9 | 1.2</td></tr>
<tr><td>Raw Vegetables</td><td>1.2 | 1.2</td><td>3.7 | 2.8</td><td>5.3 | 3.7</td><td>10.3 | 9.3</td><td>19.9 | 16.5</td><td>18.4 | 21.5</td><td>24.3 | 27.1</td><td>16.8 | 17.8</td></tr>
<tr><td>Cooked Vegetables</td><td>0.6 | 0.6</td><td>2.2 | 1.2</td><td>4.0 | 3.1</td><td>5.6 | 5.3</td><td>18.4 | 15.9</td><td>20.9 | 21.2</td><td>30.2 | 30.5</td><td>18.1 | 22.1</td></tr>
<tr><td>White Meat</td><td>13.1 | 14.0</td><td>2.5 | 1.9</td><td>4.7 | 5.3</td><td>18.1 | 17.4</td><td>40.5 | 39.3</td><td>16.8 | 16.8</td><td>3.1 | 4.4</td><td>1.2 | 0.9</td></tr>
<tr><td>Red Meat</td><td>16.2 | 16.5</td><td>10.9 | 8.4</td><td>11.2 | 11.5</td><td>31.5 | 33.3</td><td>25.9 | 26.2</td><td>4.0 | 3.7</td><td>0.3 | 0.3</td><td>-</td></tr>
<tr><td>Processed Meat</td><td>21.2 | 22.1</td><td>16.5 | 15.6</td><td>11.5 | 8.7</td><td>20.9 | 19.3</td><td>15.0 | 19.6</td><td>9.3 | 8.7</td><td>5.6 | 5.6</td><td>- | 0.3</td></tr>
<tr><td>Plant-Based Meat Alternatives</td><td>48.0 | 49.2</td><td>16.2 | 14.6</td><td>9.3 | 10.0</td><td>8.1 | 7.8</td><td>8.7 | 7.5</td><td>6.2 | 7.2</td><td>2.8 | 3.1</td><td>0.6 | 0.6</td></tr>
<tr><td>White Fish</td><td>14.3 | 1.3</td><td>8.1 | 7.5</td><td>12.5 | 11.2</td><td>32.1 | 32.1</td><td>29.0 | 30.8</td><td>3.4 | 4.7</td><td>0.6 | 0.6</td><td>-</td></tr>
<tr><td>Blue Fish</td><td>11.5 | 11.8</td><td>9.7 | 6.9</td><td>13.1 | 13.1</td><td>34.3 | 31.8</td><td>28.0 | 31.5</td><td>2.8 | 4.0</td><td>0.6 | 0.9</td><td>-</td></tr>
<tr><td>Legumes</td><td>0.9 | 0.9</td><td>5.6 | 3.7</td><td>10.6 | 7.2</td><td>27.4 | 27.1</td><td>36.1 | 40.2</td><td>13.7 | 14.6</td><td>5.3 | 5.3</td><td>0.3 | 0.9</td></tr>
<tr><td>Eggs</td><td>3.7 | 3.4</td><td>1.6 | 1.6</td><td>4.4 | 4.7</td><td>25.9 | 19.0</td><td>44.9 | 43.9</td><td>14.0 | 20.9</td><td>4.7 | 5.6</td><td>0.9 | 0.9</td></tr>
<tr><td>Milk</td><td>23.7 | 25.2</td><td>9.0 | 6.2</td><td>4.4 | 4.0</td><td>3.1 | 5.3</td><td>6.9 | 5.6</td><td>6.9 | 7.5</td><td>35.5 | 32.7</td><td>10.6 | 13.4</td></tr>
<tr><td>Plant-Based Milk Alternatives</td><td>51.1 | 50.5</td><td>12.1 | 11.8</td><td>3.1 | 2.2</td><td>2.8 | 5.0</td><td>5.0 | 4.4</td><td>6.2 | 5.9</td><td>14.6 | 14.3</td><td>5.0 | 5.9</td></tr>
<tr><td>Yogurt and Kefir</td><td>14.3 | 13.7</td><td>7.5 | 6.5</td><td>8.7 | 6.2</td><td>9.0 | 6.9</td><td>23.4 | 20.9</td><td>12.5 | 19.9</td><td>21.5 | 22.1</td><td>3.1 | 3.7</td></tr>
<tr><td>Cheese</td><td>8.7 | 8.4</td><td>4.4 | 4.4</td><td>4.4 | 4.4</td><td>12.5 | 10.6</td><td>26.5 | 25.9</td><td>23.4 | 26.5</td><td>17.1 | 16.5</td><td>3.1 | 3.4</td></tr>
</table>

White Flour Bread	12.8 ǀ 13.7	12.8 ǀ 9.0	5.9 ǀ 7.8	11.2 ǀ 12.8	16.5 ǀ 18.1	15.6 ǀ 11.8	19.0 ǀ 19.0	5.9 ǀ 7.8
Whole-Wheat Bread	15.6 ǀ 19.6	14.0 ǀ 11.2	9.0 ǀ 8.4	11.2 ǀ 9.3	15.3 ǀ 16.8	14.3 ǀ 15.0	18.7 ǀ 17.4	1.9 ǀ 2.2
Rice and White Flour Pasta	2.5 ǀ 2.5	3.1 ǀ 1.9	5.3 ǀ 6.2	15.6 ǀ 17.8	37.7 ǀ 38.0	24.3 ǀ 22.7	9.3 ǀ 8.7	2.2 ǀ 2.2
Brown Rice and Whole-Wheat Pasta	25.2 ǀ 25.9	16.8 ǀ 14.0	9.3 ǀ 10.9	17.4 ǀ 17.4	17.1 ǀ 18.7	10.0 ǀ 10.3	2.8 ǀ 2.2	1.2 ǀ 0.6
Pizza	7.5 ǀ 12.1	29.3 ǀ 23.7	24.6 ǀ 24.0	35.8 ǀ 36.8	2.5 ǀ 3.1	0.3 ǀ 0.3	-	-
Industrial Pastries	23.1 ǀ 22.1	25.9 ǀ 27.1	17.8 ǀ 16.2	12.5 ǀ 14.3	11.8 ǀ 9.3	5.3 ǀ 6.5	2.8 ǀ 4.0	0.9 ǀ 0.3
Homemade Pastries	27.7 ǀ 15.0	38.3 ǀ 20.6	18.1 ǀ 25.5	10.0 ǀ 19.3	4.7 ǀ 14.0	0.9 ǀ 4.0	0.3 ǀ 1.2	- ǀ 0.3
Nuts	4.7 ǀ 6.5	15.3 ǀ 11.2	10.6 ǀ 11.2	12.5 ǀ 14.0	21.2 ǀ 18.7	13.4 ǀ 15.0	18.7 ǀ 18.1	3.7 ǀ 5.3
Chocolate	6.9 ǀ 6.9	13.7 ǀ 11.8	13.4 ǀ 8.4	11.8 ǀ 15.0	18.7 ǀ 14.6	13.7 ǀ 16.2	19.3 ǀ 22.1	2.5 ǀ 5.0
Salty Snacks	20.2 ǀ 20.9	29.3 ǀ 21.5	15.0 ǀ 13.7	22.7 ǀ 21.8	11.5 ǀ 17.1	0.9 ǀ 4.9	0.3 ǀ 0.9	-
Soft Drinks	51.7 ǀ 54.8	25.9 ǀ 20.9	9.3 ǀ 9.3	5.9 ǀ 7.8	3.7 ǀ 3.4	1.9 ǀ 2.5	1.2 ǀ 0.9	0.3 ǀ 0.3
Non-Alcoholic Beverages	2.5 ǀ 3.4	3.1 ǀ 2.5	3.1 ǀ 1.9	1.6 ǀ 1.2	2.2 ǀ 2.8	4.4 ǀ 3.4	25.5 ǀ 24.0	57.6 ǀ 60.7
Fermented Alcoholic	21.8 ǀ 39.6	20.9 ǀ 15.9	12.1 ǀ 9.3	17.4 ǀ 12.5	17.4 ǀ 10.9	6.2 ǀ 7.5	4.0 ǀ 4.0	- ǀ 0.3
High-Grade Beverages	38.9 ǀ 73.8	34.9 ǀ 16.5	17.4 ǀ 6.9	5.6 ǀ 1.9	2.2 ǀ 0.9	0.9 ǀ -	-	-

* Before Lockdown ǀ During Lockdown.
